# Microbial and host innate immune factors affecting the persistence of *Staphylococcus aureus* in cystic fibrosis airways

**DOI:** 10.3389/fcimb.2026.1797869

**Published:** 2026-06-03

**Authors:** Jacob D. Fairholm, Gavin Thomas Paul, Susan Birket, Balázs Rada

**Affiliations:** 1 Department of Infectious Diseases, College of Veterinary Medicine, The University of Georgia, Athens, GA, United States; 2 Division of Pulmonary, Allergy, and Critical Care Medicine, The University of Alabama at Birmingham, Birmingham, AL, United States

**Keywords:** biofilm, cystic fibrosis - CF, neutrophil extracellular trap, neutrophils, persistence, small-colony variant (SCV), *Staphylococcus aureus*, virulence factors

## Abstract

Chronic bacterial respiratory infections by *Staphylococcus aureus* are a hallmark of cystic fibrosis (CF) lung disease, affecting up to 80% of all people with CF by their mid-teens. *S. aureus* is able to survive and persist in the CF lung despite robust neutrophilic inflammation. As neutrophils are the immune system’s front line of defense against bacteria, the persistent nature of *S. aureus* infections in CF indicates both a defect in the ability of neutrophils to kill *S. aureus* and an enhanced ability of the bacteria to survive. *S. aureus* persistence in the CF lung is driven by both microbial and host, genetic and microenvironmental, factors. There doesn’t seem to be one unifying feature that makes *S. aureus* more virulent in the CF lung as several factors have been proposed to aide its survival. These include increased resistance to antibiotics, the ability to form small colony variants, biofilm formation, co-infection with *Pseudomonas aeruginosa*, and several virulence factors such as the accessory gene regulatory system, leukocidins, and staphylococcal protein A. A variety of host factors also affect the ability of neutrophils to kill *S. aureus* in the CF lung. Defects in the function of the cystic fibrosis transmembrane conductance regulator, the genetic cause of CF, affect phagolysosomal killing and lead to increased formation of neutrophil extracellular traps, which are less effective at killing *S. aureus*. Additionally, data suggest that factors within the CF lung microenvironment also affect neutrophilic killing of *S. aureus*. However, more research is needed to clearly identify what these environmental factors may be. This review article summarizes the current knowledge on the clinical relevance of *S. aureus* lung infections in CF, on microbial and host mechanisms promoting *S. aureus* survival in the CF lung, and on details of neutrophil-*S. aureus* interactions in CF. By understanding how *S. aureus* is able to survive in the CF lung and why neutrophils are unable to kill this bacterium, it could be possible to identify potential therapeutic targets to alleviate the consequences of *S. aureus* respiratory infection in CF.

## 1 Introduction

Cystic fibrosis (CF) is a genetic disease caused by mutations in the cystic fibrosis transmembrane conductance regulator (CFTR) gene. CFTR is an ion channel responsible for transporting chloride and other anions across the cell membrane. Over 2,000 CFTR mutations are known to cause CF but the most common is a deletion of a phenylalanine at position 508 (ΔF508), carried by about 85% of affected individuals in the United States (Grasemann and Ratjen, 2023). Mutations to CFTR result in impaired ion transport which leads to a thickening of the mucus layer, impaired mucociliary clearance and subsequent bacterial infections and inflammation.

Chronic lung infections are a hallmark of CF and are associated with decreased lung function leading to respiratory failure which is the leading cause of death in people with CF (PwCF) (Blanchard and Waters, 2019). Infections of the CF airways are often polymicrobial with *Haemophilus influenzae*, *Strenotrophomonas maltophilia*, *Achromobacter xylosoxidans*, *Burkholderia cepacian* complex members, and *Mycobacterium abscessus* being commonly isolated from the sputum and bronchoalveolar lavage fluid (BALF) of PwCF (Lipuma, 2010; Blanchard and Waters, 2019; Cystic Fibrosis Foundation, 2025). However, the two most common bacterial pathogens present in the CF lung are *Staphylococcus aureus* and *Pseudomonas aeruginosa* (Cystic Fibrosis Foundation, 2025).

Another hallmark of CF is chronic inflammation of the airways. The lungs of PwCF demonstrate an increase in proinflammatory cytokines, including interleukin- 6 (IL-6), IL-8, and tumor necrosis factor alpha (TNF-α), as well as a decrease in the anti-inflammatory cytokine IL-10 (Koehler et al., 2004). Children with CF have especially high levels of IL-1β in their lungs (Montgomery et al., 2018; Fantone et al., 2025). These cytokines, and other chemoattractants, recruit neutrophils in high numbers to the CF lung. Under healthy conditions, the role of neutrophils is to kill pathogens through phagocytosis followed by subsequent death by apoptosis and clearance by macrophages that prevents damage caused by hyper-inflammation. However, neutrophils in the CF lung environment release a large number of neutrophil-derived serine proteases, reactive oxidants, neutrophil extracellular traps (NETs) and the proinflammatory cytokines listed above (Mall et al., 2024). This excessive inflammation drives further mucociliary clearance defects and structural damage, fueling a destructive positive feedback loop. Despite heavy neutrophilic inflammation, bacterial infections persist, indicating a defect in neutrophil function in CF that could arise from 1) the CFTR mutation itself or 2) features of the CF airway environment (Patel and Nugent, 2023).

This review focuses on *S. aureus* infection in CF, it’s prevalence and adaptations to survive in the CF lung environment, as well as on factors, both host and microbial, that prevent its effective clearance by neutrophils.

## 2 Prevalence and clinical significance of *S. aureus* infection in CF

*S. aureus* is one of the first pathogens to be detected in the airways of PwCF early in life (Rumpf et al., 2021). A 2015 single-center study found that among adult CF patients, 24% were infected with *S. aureus* while 60% were infected with *P. aeruginosa* (Ahlgren et al., 2015). In 2018, over half of children with CF below the age of two were reported to be infected with *S. aureus*. That number rose to more than 80% of PwCF in their teenage years (Blanchard and Waters, 2019; Rumpf et al., 2021). According to the Cystic Fibrosis Foundation’s patient registry annual data report in 2024, 48% of all PwCF who provided samples had at least one positive culture for methicillin-sensitive *S. aureus* (MSSA) (Cystic Fibrosis Foundation, 2025). *S. aureus* infection early in life will become chronic in about one third of PwCF (Thornton and Parkins, 2023). However, the prevalence of *S. aureus* infection falls as PwCF enter adulthood, with *P. aeruginosa* taking over as the most common respiratory pathogen. One 2022 study found that among PwCF under the age of 18 that were homozygous for the ΔF508 mutation, 55% had tested positive for *S. aureus* in the United States while only 10% tested positive in the United Kingdom (Schlüter et al., 2022). More significantly, methicillin-resistant *S. aureus* (MRSA) also represents a growing problem in CF, with nearly 20% of all PwCF in the United States having positive cultures in 2024 (Cystic Fibrosis Foundation, 2025). However, the prevalence of MRSA in Canada in 2016 was only 6.4% and 2.6% in Australia in 2015 (Akil and Muhlebach, 2018). This suggests that the prevalence of *S. aureus* infections in PwCF may also be dependent on the geographic location. Thus, *S. aureus* infection represents a significant clinical challenge in CF, particularly in the first two decades of life.

While *S. aureus* is the most common pathogen in the airways of young PwCF, its precise clinical impact on long-term lung function decline and exacerbation rates, especially in older patients, has been a subject of ongoing debate. Several studies support the association of *S. aureus* airway infections with worse lung disease and clinical outcomes in PwCF. In PwCF (age > 6 years) with persistent *S. aureus* cultures, high bacterial density in throat cultures was associated with a steeper lung function decline (Ahlgren et al., 2015). Small colony variants (SCVs) of *S. aureus* are also common among CF clinical isolates. The presence of *S. aureus* SCVs was also associated with worse overall lung function (Ahlgren et al., 2015). Infection with *S. aureus* SCVs was independently associated with a greater decrease in lung function during the study in a pediatric CF cohort, even after adjusting for *P. aeruginosa* and MRSA (Wolter et al., 2013). Early chronic infection with MSSA before the age of 4 was associated with poorer lung function and a 79% increased risk of lung exacerbations by age 8 (Sunman et al., 2022). In a Brazilian cohort of young PwCF (age: 8–23 years), patients infected with *S. aureus* had a lower forced vital capacity (FVC), forced expiratory volume in one second (FEV_1%_, a common method to measure lung function) and a higher frequency of pulmonary exacerbations compared to those infected with *P. aeruginosa* (Kussek et al., 2022). One systemic review found that FEV_1%_ was nearly 17% lower in PwCF colonized by SCVs than those colonized by prototypical variants of *S. aureus*, indicating worse lung function (Ryan et al., 2023).

Other studies, however, report no significant association between *S. aureus* respiratory infection and lung disease in PwCF. In a retrospective cross-sectional study of 84 adult PwCF, those with *S. aureus* infection alone (in the absence of *P. aeruginosa*) experienced fewer pulmonary exacerbations and lower C-reactive protein levels than those infected with *P. aeruginosa* (Ahlgren et al., 2015). Another report examining the relationship between lung function and microbial communities in PwCF found that among individuals with decreasing lung function, *P. aeruginosa* was more likely to predominate while *S. aureus* was less likely to be dominant (Thornton et al., 2023). This same report found that among PwCF where *S. aureus* was the dominant bacterial infection, there was a 10% decrease in survival rate, although this was not statistically significant (Thornton et al., 2023). In an analysis of *S. aureus* and *P. aeruginosa* infections in adult PwCF, patients with single *S. aureus* infection (no *P. aeruginosa*) had a less severe clinical condition than those with *P. aeruginosa* (Briaud et al., 2020). However, patients with co-infection presented with a higher frequency and length of hospitalization and more exacerbations than those only infected with *S. aureus* (Briaud et al., 2020). In a prospective, longitudinal study, the presence of specific *S. aureus* virulence genes (from a panel of 25) was not associated with clinical deterioration (FEV_1%_ decline or exacerbation) (Lange et al., 2020).

Overall, *S. aureus* is the most prevalent pathogen in young children with CF and is often linked to early inflammation and decline in lung function. In contrast, its impact in adults, especially in the absence of *P. aeruginosa*, may be less severe. Specific features of *S. aureus* infection like SCVs, high bacterial density, co-infection with other pathogens (like *P. aeruginosa* or *Stenotrophomonas maltophilia*), antibiotic resistance (MRSA), or any combination of these are key independent factors associated with more rapid decline in lung function and worse outcomes in PwCF. Thus, there are mixed recommendations for staphylococcal treatments in CF worldwide. In the United Kingdom, Germany, and Australia antibiotics are recommended for all CF children, while in the United States antibiotic usage is only recommended during disease exacerbations (Akil and Muhlebach, 2018). This is because the prophylactic use of anti-staphylococcal therapy was found to increase the risk of *P. aeruginosa* infection (Stutman et al., 2002). Therefore, the Cystic Fibrosis Foundation determined that the risk of earlier or more frequent *P. aeruginosa* infection did not outweigh the benefits of reduced rates of *S. aureus* infections (Flume et al., 2007).

Several studies have sought to examine the relationships of *S. aureus* strains isolated from PwCF. Their goal was to better understand how *S. aureus* is transmitted from patient to patient and to identify potential characteristics unique to CF-associated *S. aureus* isolates. One study collected 64 *S. aureus* isolates from 50 patients of all ages from two different CF clinics in the United States (Bernardy et al., 2020). They found that the isolates grouped into 8 different phylogenetically distinct clonal clusters, none of which were closely related enough to suggest transmission from one patient to another (Bernardy et al., 2020). They also found that, despite the conventional hypothesis, *S. aureus* does not lose virulence during CF infection, as a majority of isolates expressed common virulence factors including antibiotic-resistance genes, alpha and beta toxin genes, and the ability to produce polysaccharides (Bernardy et al., 2020). Another study from 2020 performed whole genome sequencing on 1,382 *S. aureus* isolates collected from 246 children with CF from 5 different CF centers across the United States (Long et al., 2021). They found that the sequences of the isolates did not group according to the geographic location of the patients, suggesting that *S. aureus* diversity is not restricted to the location of the clinic (Long et al., 2021). Unlike the study above, this report found that there was evidence of transmission between patients, particularly between siblings and PwCF with close contact attending the same clinic, and that both community- and health care-associated transmission was possible (Long et al., 2021). Interestingly, when multiple strains were isolated from a single patient, those isolates most often (81%) came from different lineages, suggesting multiple concurrent infections with different CF strains rather than the evolution of one initial strain (Long et al., 2021). Additionally, these concurrent strains may have differing susceptibility to antibiotics (Long et al., 2021). Finally, they found that CF isolates of *S. aureus* did not mutate faster than non-CF isolates, suggesting that adaptation to the CF airway environment is not due to hypermutation as previously thought (Long et al., 2021). These two studies show that CF clinical isolates of *S. aureus* do not share much genetic similarity from patient to patient as genetic diversity among isolates is high across large populations of PwCF, although some localized transmission from patient to patient is possible. Additionally, the size of the patient cohort and number of isolates obtained is important to consider when attempting to draw conclusions about the genetic diversity and relatedness of *S. aureus* isolates. Overall, while *S. aureus* is one of the most common lung infections in PwCF and leads to poorer health outcomes, there is not one, well-defined feature of *S. aureus* that makes it more or less likely to establish infection in the CF lung.

## 3 Microbial factors leading to *S. aureus* persistence in CF

*S. aureus* is a gram-positive, facultative anaerobe bacterium that is a common commensal organism of the skin and nares of people without CF (Goss and Muhlebach, 2011). In fact, roughly 30-50% of healthy individuals are colonized either intermittently or chronically with *S. aureus* (Goss and Muhlebach, 2011; Brown et al., 2014). One study found that while only 25% of individuals involved were considered carriers of *S. aureus*, 100% of the 996 individuals had detectable levels of both anti-staphylococcal IgA and IgG antibodies in their serum, suggesting near universal exposure to the bacterium (Meyer et al., 2021). However, some of the detectable anti-staphylococcal antibodies may be due to indirect exposure to the bacterium or cross-reactivity of other antibodies. In people without CF, colonization of the anterior nares with *S. aureus* is the leading cause of bacteremia, however non-colonized individuals are more likely to die from *S. aureus* bacteremia than colonized individuals, suggesting a certain degree of control by the immune system in people with *S. aureus* colonization (Wertheim et al., 2004). Other risk factors for *S. aureus* infection include old age, immunodeficiencies (especially those due to HIV infection), injection drug use, and ethnicity, with *S. aureus* bacteremia being more common among people of color (Tong et al., 2015).

Infection with *S. aureus*, especially in PwCF, is aided by the implementation of several key virulence factors. Among these virulence factors are toxins such as the leukocytolytic toxin Panton-Valentine leucocidin, the acquisition of antibiotic resistance genes such as *mecA* which leads to the development of methicillin resistance, as well as microbiological adaptations such as the formation of SCVs and biofilms (Akil and Muhlebach, 2018; Blanchard and Waters, 2019; Sheykhsaran et al., 2024). The next sections will focus on the role of antibiotic resistance, SCVs, biofilms, and virulence toxins in aiding *S. aureus* persistence in the CF lung.

### 3.1 Antibiotic resistance

**Figure 1:**
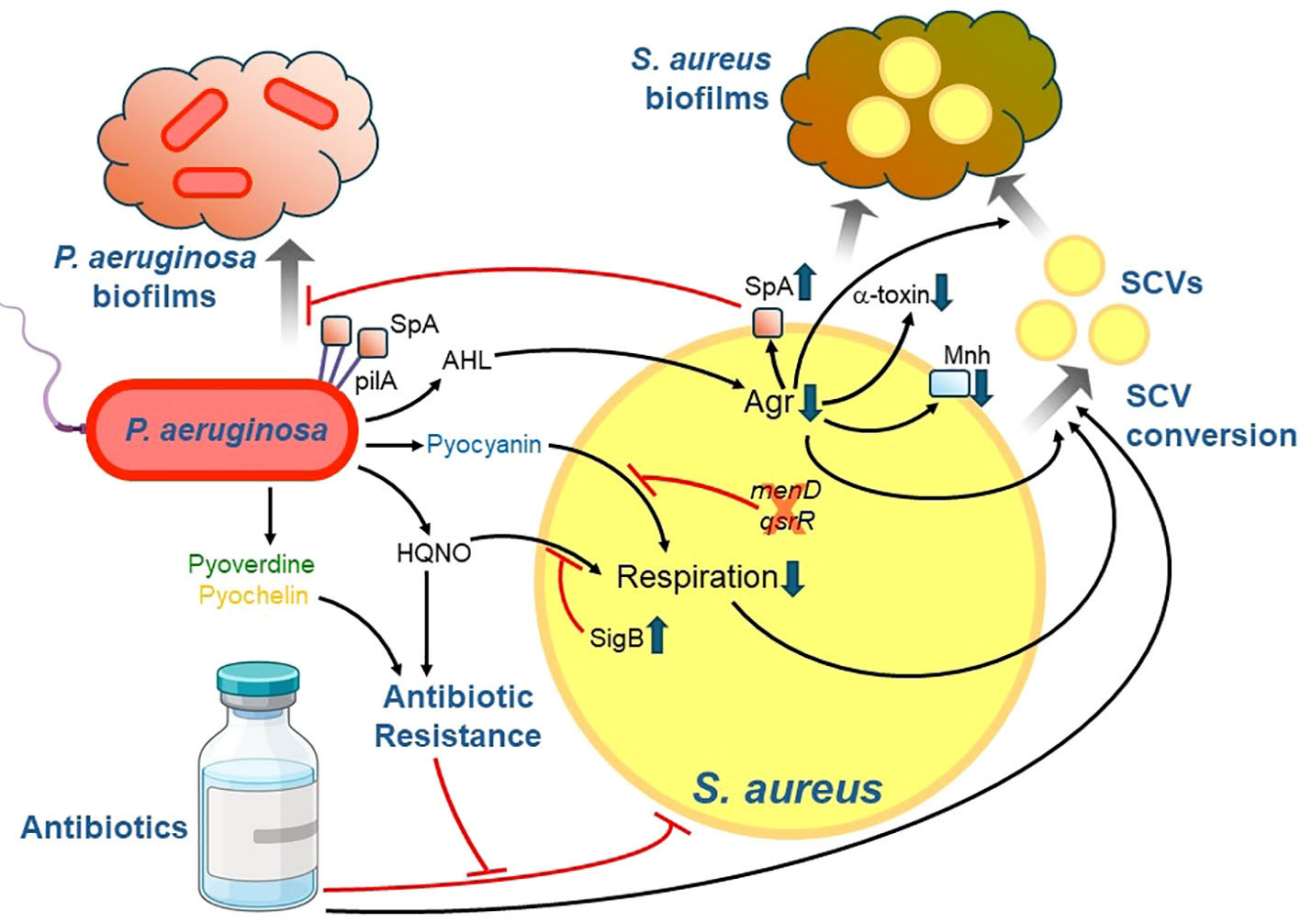
Interactions between *S. aureus* and *P. aeruginosa* in the CF lung. *S. aureus* and *P. aeruginosa* are often involved in polymicrobial lung infections in PwCF. In low doses, anti-staphylococcal factors such as pyoverdine, pyochelin, and HQNO can lead to increased antimicrobial resistance in *S. aureus*. HQNO can also induce SCV and biofilm formation through SigB signaling. SCVs and biofilms both increase antibiotic resistance in *S. aureus*. AHLs produced by *P. aeruginosa* can lead to the down-regulation of Agr, resulting in increased SCV formation and SpA production. SpA can inhibit *P. aeruginosa* biofilm production by binding to PsL and PilA. Pyocyanin generated by *P. aeruginosa* inhibits respiration in *S. aureus* and therefore promotes SCV conversion. Black arrows indicate stimulatory mechanisms while red lines represent inhibitory effects. Blue vertical arrows indicate whether the mechanism is up- or down-regulated in CF. SCV, small colony variants; AHL, N-acylhomoserine lactones; HQNO, 4-hydroxy-2-heptylquinoline-N-oxide.

As with other conditions, antibiotic resistance of *S. aureus* is a growing concern in CF. Among CF isolates of *S. aureus*, erythromycin and clindamycin have the highest levels of resistance (Xu et al., 2024). During pulmonary exacerbations, 80% of *S. aureus* isolates were resistant to azithromycin and erythromycin, 52% were resistant to ciprofloxacin, and 20% were methicillin-resistant (Erfanimanesh et al., 2022). However, the most concerning antibiotic resistance in *S. aureus* is methicillin resistance. MRSA infections now affect nearly 20% of all PwCF (Cystic Fibrosis Foundation, 2025). Resistance to methicillin is mediated through an altered penicillin binding protein, PBP-2a, encoded by the *mecA* gene, which has a poor affinity for beta-lactam antibiotics (Raygada and Levine, 2009). The *mecA* gene is carried on the staphylococcal cassette chromosome, a mobile genetic element of which there are 14 known types (Akil and Muhlebach, 2018; Uehara, 2022). In a multi-center study of nearly 20,000 PwCF, those patients who acquired MRSA were most often younger (mean age of 15 years), had pancreatic insufficiencies, and were previously infected with *P. aeruginosa* and MSSA (Dasenbrook et al., 2010). MRSA infection associated with increased number of acute pulmonary exacerbations (Dasenbrook et al., 2010). Additionally, the risk of death was increased 1.27 times when MRSA was detected versus when MRSA was absent (Dasenbrook et al., 2010). Thus, MRSA infection is associated with worse clinical outcomes in PwCF. Interestingly, hypoglycemia, which is common in CF as CFTR deficiency also affects the pancreas, may lead to increased antibiotic resistance (Hughes et al., 2025). When grown on air-liquid interface with CF bronchial epithelial cells, *S. aureus* developed increased resistance to rifampicin under hypoglycemic conditions (Hughes et al., 2025). CF clinical isolates of MSSA gained increased resistance to other beta-lactam antibiotics as the concentration of bacteria increased, a process known as the inoculum effect (Svishchuk et al., 2023). This is due to the production of beta-lactamase which normally targets penicillin but can have weak effects on other beta-lactams at high concentrations, such as those found in CF airways (Svishchuk et al., 2023). In order to identify how antibiotic resistance changes over time, clinical isolates of *S. aureus* from pediatric PwCF were isolated at early and late time points (Ding et al., 2023). Most early isolates were exclusively resistant to penicillin, which did not change with the later isolates (Ding et al., 2023). However, a few isolates lost resistance to penicillin while others gained additional resistance to rifampicin (Ding et al., 2023). The spread of antibiotic resistance in CF isolates of *S. aureus* could be aided by bacteriophages as one study found that 32.4% of CF sputum samples harbored phages containing antimicrobial resistance genes (Brown-Jaque et al., 2018). Beta-lactamase genes were most often found in sputum phages while *mecA* was rarely found (Brown-Jaque et al., 2018). Antibiotic resistance in *S. aureus* can also arise in association with other phenotypic changes, including SCV and biofilm formation (**Figure 1**).

### 3.2 Small colony variants

SCVs have several morphological differences compared to normal colony variants (NCVs), including smaller colony size (up to 10-fold as small) originating from slower growth, typically taking 48–72 hours to become visible on agar plates (Zhou et al., 2022; Sheykhsaran et al., 2024). On blood agar plates, SCVs are small, less than 0.2 mm in diameter, non-pigmented, and non-hemolytic, making them difficult to detect (Quie, 1969; Becker, 2023). On mannitol salt agar plates, SCVs are non-mannitol fermenting (Carris et al., 2025). Currently, there are no consensus recommendations for medical microbiology labs to detect *S. aureus* SCVs in CF (Saiman et al., 2024). However, *S. aureus* SCVs were identified from chronic wound samples based on the morphological differences of the colonies on blood agar and mannitol salt agar plates described above followed by species determination by MALDI-TOF or BD Phoenix Gram-positive identification panels (Carris et al., 2025). In general, SCVs arise due to alterations in metabolism or changes in global gene regulatory systems typically as a result of growth in sub-optimal conditions (Quie, 1969; Mapar et al., 2025). The genetic mutations in metabolic processes that lead to SCV formation result in auxotrophy, an inability to produce a particular compound required for growth (Zhou et al., 2022; Sheykhsaran et al., 2024). In CF, *S. aureus* SCV strains are most commonly auxotrophic for thymidine, menadione, and hemin (Masoud-Landgraf et al., 2016; Zhou et al., 2022). Persistent use of antibiotics has been identified as a common selector for SCV formation, especially as many SCV strains are antibiotic-resistant (**Figure 1**) (Zhou et al., 2022; Sheykhsaran et al., 2024). One systematic review of the risk factors for SCV occurrence in PwCF showed that prior use of trimethoprim sulfamethoxazole was 68% more likely to lead to the occurrence of SCVs than that of NCVs (Ryan et al., 2023).

The occurrence of SCVs is common in PwCF. One study in Austria found that SCVs could be isolated from 9.4% of CF individuals testing positive for *S. aureus* (Masoud-Landgraf et al., 2016). Another study in Canada found that 72% of CF patients that tested positive for *S. aureus* had SCVs (Millette et al., 2023). However, this study included far fewer participants than the Austrian study. Another multi-center study of children with CF conducted in the United States determined that 26% of patients tested positive for *S. aureus* SCVs (Wolter et al., 2019). Therefore, the occurrence of SCV of *S. aureus* is common but varies based on the location and the size of study.

SCVs of *S. aureus* may contribute to the persistent and chronic nature of *S. aureus* lung infection in PwCF. One study from 1998 found that 26 out of 78 PwCF harbored *S. aureus* SCV infections (Kahl et al., 1998). Additionally, SCVs were further isolated for up to 31 months following initial characterization from 19 out of 26 patients (Kahl et al., 1998). Antibiotic resistance may contribute to SCV persistence. 38% of SCV CF clinical isolates from the Canadian study were resistant to at least one aminoglycoside (Millette et al., 2023). Additionally, it was found that SCVs were more resistant to both ciprofloxacin and vancomycin, compared to NCVs isolated from the same CF individual (Millette et al., 2023). SCVs were also found to be more likely to acquire resistances to gentamicin and ciprofloxacin after multiple passages at sub-inhibitory concentrations, as well as increased rates of mutation upon exposure to rifampicin, showing that SCVs more readily adapt to antibiotic pressure than NCVs (Millette et al., 2023).

Another mechanism that may contribute to SCV persistence is their increased ability to survive within host epithelial cells and phagocytes. Intracellular survival of *S. aureus* can be attributed to the expression of bacterial factors, primarily those associated with the accessory gene regulator (Agr) system, that prevent the fusion of the lysosome with the phagosome as well as aid bacterial escape into the cytoplasm (Löffler et al., 2014). In one study, CFTR-deficient epithelial cells were shown to be more likely to be infected with *S. aureus* than epithelial cells with functioning CFTR (Mitchell et al., 2011). Additionally, SCVs were better able to infect both CF and non-CF epithelial cells than NCVs (Mitchell et al., 2011). Another study showed that intracellular infection of endothelial cells with a NCV of *S. aureus* significantly increased the expression of proinflammatory cytokines compared to intracellular infection with a SCV (Tuchscherr et al., 2010). Additionally, SCV colonies were recovered at consistent levels throughout four days of intracellular infection, whereas the number of NCV colonies recovered was reduced over time (Tuchscherr et al., 2010). This is likely due to the fact that SCVs express less Agr genes, particularly alpha-toxin, which has been shown to induce inflammation during intracellular infection (Tuchscherr et al., 2010; Mitchell et al., 2011; Löffler et al., 2014). These findings were corroborated by an additional study that found that CF clinical SCV isolates expressed less Agr genes and displayed increased intracellular survival than both NCVs and non-CF SCVs (Moisan et al., 2006).

The emergence of SCVs of *S. aureus* may be associated with co-infections with *P. aeruginosa*, a common occurrence in PwCF. One report found that after a 5 day co-culture of *S. aureus* with *P. aeruginosa*, the majority of recovered *S. aureus* colonies were SCVs (Hoffman et al., 2006). This was likely due to an anti-gram-positive compound produced by *P. aeruginosa* known as 4-hydroxy-2-heptylquinoline-*N*-oxide (HQNO) (**Figure 1**). When HQNO was incubated with wild-type *S. aureus*, SCVs emerged (Hoffman et al., 2006). However, when HQNO-deficient *P. aeruginosa* was co-cultured with *S. aureus*, fewer SCVs were recovered (Hoffman et al., 2006). SCVs also arose following the formation of co-species biofilms in a chronic wound infection model (Gounani et al., 2020). This was corroborated by another co-species biofilm study that also found a decrease in *S. aureus* colony size when grown in the presence of *P. aeruginosa* as compared to those grown in pure culture (Kumar and Ting, 2015). The formation of *S. aureus* SCVs in co-cultures with *P. aeruginosa* is likely a survival mechanism as *P. aeruginosa* produces several anti-staphylococcal compounds including HQNO and pyocyanin, leading to the selection of resistant SCVs over susceptible NCVs (Biswas et al., 2009). Pyocyanin is a redox-active virulence factor of *P. aeruginosa* (Rada and Leto, 2013) that inhibits respiration in *S. aureus*, forces it to gain energy from fermentation and also reduces its biofilms (Noto et al., 2017; Kamer et al., 2023). *S. aureus* can gain resistance to pyocyanin by mutations emerging in the *qsrR* and *menD* genes, indicating the relevance of menadione auxotrophy and repression of quinone detoxification in pyocyanin resistance (Noto et al., 2017) (**Figure 1**).

*S. aureus* SCV formation has also been associated with increased biofilm formation. One study found that CF SCV isolates of *S. aureus* produced significantly more biofilms than their NCV counterparts (Millette et al., 2023). Interestingly, another study found HQNO exposure to NCVs caused an increase in biofilm production but SCVs did not increase biofilm production when exposed to HQNO (Mitchell et al., 2010; Roman-Rodriguez et al., 2025). However, these SCVs already produced more biofilms compared to the NCVs at baseline, leading the researchers to conclude that sustained HQNO exposure leads to an increase in biofilm production by converting NCVs to SCVs (Mitchell et al., 2010). The effect of HQNO on SCV and biofilm production is dependent on the alternate sigma factor B (SigB), which controls the *S. aureus* response to stress, as *S. aureus* strains lacking SigB showed reduced SCV and biofilm formation (**Figure 1**) (Mitchell et al., 2010).

Overall, it seems as though SCVs increase the ability of *S. aureus* infections to persist in the CF lung through several different mechanisms, including increased antibacterial resistance, improved intracellular survival and immune avoidance, improved resistance to *P. aeruginosa* competition, and increased biofilm formation.

### 3.3 Biofilms

Biofilms represent another factor leading to the persistence of *S. aureus* infections in the CF lung. *S. aureus* biofilms are of an extracellular matrix composed of polysaccharides, proteins, and extracellular DNA (eDNA), although the exact composition varies greatly according to strain, infection site, time of infection, and other conditions (Parastan et al., 2020; Bowden et al., 2024; Wu et al., 2024). The two main polysaccharides involved in *S. aureus* biofilm formation are the polysaccharide intracellular adhesin (PIA) and the capsular polysaccharide (CP) (Riquelme et al., 2020). PIA may be the predominate polysaccharide in CF biofilms as one study found that 80-90% of human cells infected with CF isolates of *S. aureus* were positive for PIA while less than 10% were positive for CP (McKenney et al., 1999). Although traditionally considered to be associated with attachment to a surface, biofilms can also take the form of free-floating, three-dimensional aggregates which are more common in the CF airway (Jean-Pierre et al., 2022). Biofilms are typically detected by the binding of dyes such as crystal violet or the detection of polysaccharides like PIA (Allkja et al., 2020; Bernardy et al., 2020). The ability of *S. aureus* to form biofilms is common among CF isolates. One study found that 96.8% of CF isolates were able to form biofilms while only 47.4% of non-CF *S. aureus* isolates where able to do so (Cakır Aktas et al., 2013). Another study of pediatric PwCF found that 67% of *S. aureus* clinical isolates were able to produce biofilms (Kodori et al., 2021). A more recent study investigating the mucoid phenotype of *S. aureus* in PwCF reported that between 67.5% to 72.9% of mucoid *S. aureus* isolates produced biofilms (Rumpf et al., 2025). Chronic infection can lead to increased biofilm production as one study found that isolates from late infection were more likely to develop biofilms than earlier isolates (Tan et al., 2019). This is further corroborated by another study which analyzed the adaptation of *S. aureus* isolates taken from PwCF over four years and found an increase in biofilm production by later isolates (Gabryszewski et al., 2019). This was in part due to metabolic adaptations, as the later CF isolates displayed increased expression of *fumC*, an enzyme involved in the conversion of fumarate to malate (Gabryszewski et al., 2019). However, the ability of *S. aureus* to form biofilms is not restricted to chronic infection as one study reported that 27% of CF isolates studied formed biofilms after just 4 hours of incubation in artificial CF sputum medium (Boudet et al., 2021). Therefore, there are variations in the ability of CF clinical isolates of *S. aureus* to produce biofilms, but it is a common occurrence.

Biofilm formation is likely beneficial to the survival of *S. aureus* in the CF lung environment by increasing resistance to the immune system and antibiotics. Antibiotic susceptibility is reduced among bacteria within biofilms due to the physical barrier created by the extracellular matrix which often reduces the ability of the drug to penetrate and to make contact with the bacteria or can degrade the antimicrobial compounds (Liu et al., 2024). Additionally, the relatively high number of bacteria within the biofilm reduce the susceptibility of *S. aureus* to various beta-lactams due to the inoculum effect (Dingle et al., 2022; Liu et al., 2024). Among pediatric patients with *S. aureus* infections, 85.6% of MRSA isolates produced biofilms while only 54.2% of MSSA isolates formed biofilms (Kadkhoda et al., 2020). However, this study was not specific to *S. aureus* infections in CF. The minimal inhibitory concentration of vancomycin increased against several CF MRSA isolates when grown in biofilms versus planktonic forms (Britt et al., 2020). One study found that antibiotics were less effective against both MSSA and MRSA biofilms when grown in an artificial CF sputum medium to mimic the CF lung environment, than when the biofilms were grown in a non-CF-like environment (Iglesias et al., 2019). This indicates that the combination of biofilm formation and the CF environment leads to increased antibiotic resistance in *S. aureus* isolates. However, one study reported that *S. aureus* strains adapted to the CF lung environment after years of chronic infection still demonstrated susceptibility to the fifth-generation, broad-spectrum cephalosporin antibiotic, ceftaroline (Boudet et al., 2021). The close proximity of individual bacteria within a biofilm may also lead to a rise in antibiotic resistance due to horizontal gene transfer of resistance plasmids (Savage et al., 2013). Exposure of *S. aureus* to *P. aeruginosa* in polymicrobial biofilms can also increase antibiotic resistance as exposure of *S. aureus* to HQNO and the *P. aeruginosa* siderophores pyoverdine and pyochelin increased *S. aureus* resistance to vancomycin (**Figure 1**) (Orazi and O’Toole, 2017). *S. aureus* grown in co-culture with a HQNO-deficient *P. aeruginosa* was also demonstrated to be more susceptible to tobramycin than those co-cultured with wild-type *P. aeruginosa*, providing additional evidence to the promotion of *S. aureus* antibiotic resistance by HQNO (Barraza and Whiteley, 2021). However, interestingly, *S. aureus* grown in monoculture was more resistant to tobramycin than both *P. aeruginosa* co-culture conditions (Barraza and Whiteley, 2021). Therefore, while *S. aureus* biofilm formation leads to antibiotic resistance, the exact effect of polymicrobial biofilms with *P. aeruginosa*, and more specifically HQNO exposure, may be specific to the antibiotics used.

**Figure 2:**
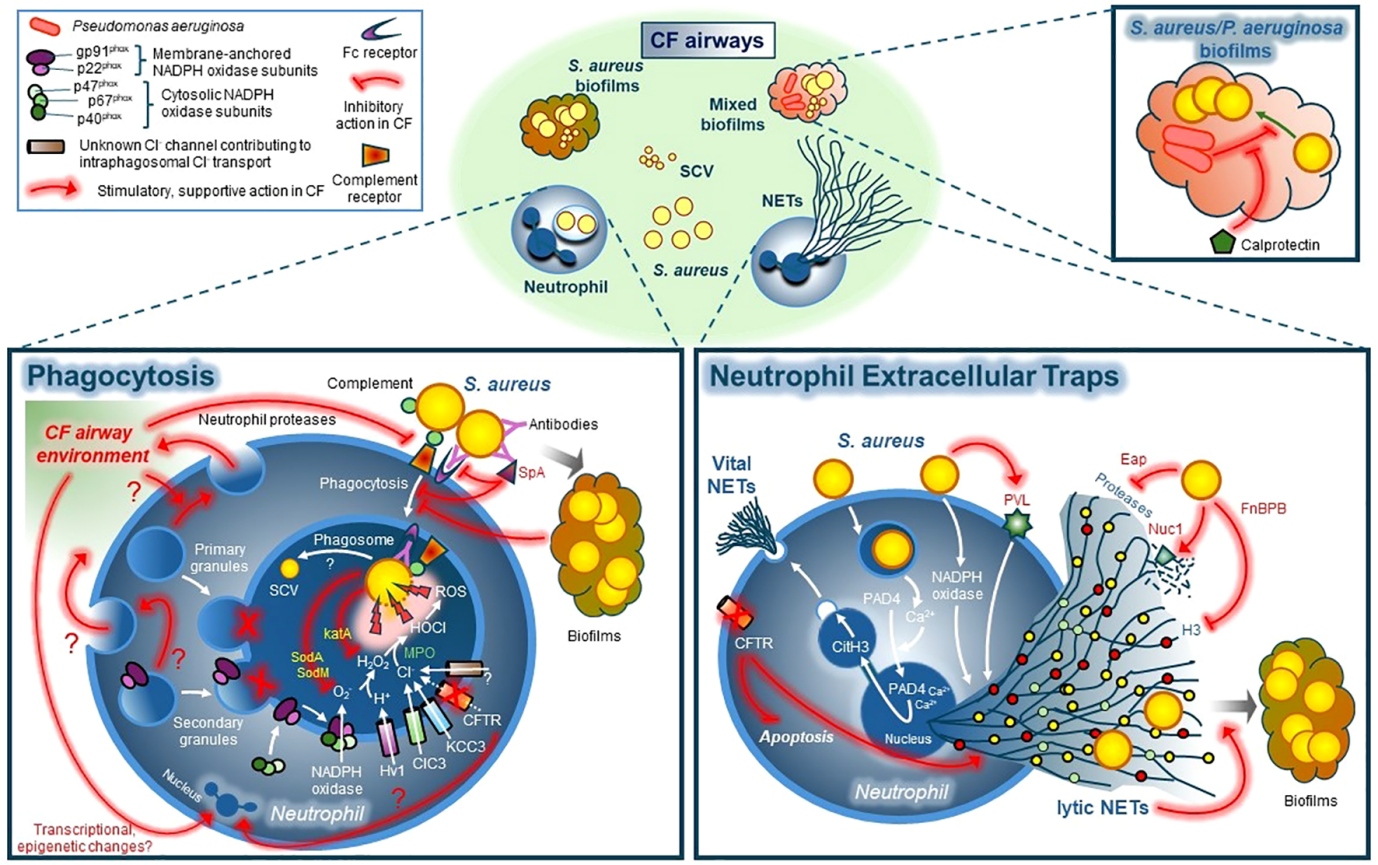
Proposed mechanisms by which *S. aureus* avoids neutrophilic killing in CF airways. The CF airway environment is very complex and hosts *S. aureus* in several forms and compartments. Normal-size colonies, small-colony variants (SCV) of the bacterium, *S. aureus* biofilms and mixed biofilms with *P. aeruginosa* have all been reported. Neutrophils are present but fail to eliminate *S. aureus* in the CF respiratory tract. In healthy individuals, neutrophils mainly kill *S. aureus* by oxidative, intracellular mechanisms that includes bacterial attachment to the neutrophil surface, engulfment into the phagosome, fusion of the phagosome with neutrophil granules, delivery of the granule cargo into the phagolysosome lumen, assembly of the NADPH oxidase, intraphagosomal production of reactive oxygen species (ROS) and ROS-mediated killing of *S. aureus*. CF neutrophils, however, fail to kill *S. aureus* that can be caused by several proposed mechanisms. CFTR deficiency leads to delayed chloride accumulation in the phagosome and impaired HOCl-mediated bacterial killing. The staphylococcal protein A (SpA) binds to IgG on the bacterial surface and inhibits FcR-mediated binding and uptake of S. aureus into neutrophils. *S. aureus* forms biofilms that inhibits phagocytosis but could also support formation of neutrophil extracellular traps (NETs). Phagosomal ROS can be quenched by bacterial antioxidant enzymes (SodA, SodM, KatA) and could also trigger *S. aureus* conversion to SCVs. The CF airway environment has also been described to hijack primary granules from fusing with the phagosome and to aim them to the plasma membrane instead. The CF lung content has been proposed to trigger transcriptional changes in neutrophils. Neutrophil-derived proteases can digest surface receptors of neutrophils, yielding impaired bacterial recognition and uptake. *S. aureus* has been described to induce NET release in neutrophils by at least two distinct mechanisms. First, *S. aureus* triggers a rapid, viable form of NET extrusion following phagocytosis that is dependent on peptidylarginine deiminase 4 (PAD4) and calcium, is independent of the NADPH oxidase and leads to citrullinated histone formation and chromatin expulsion without plasma membrane rupture via a proposed vesicular mechanism of NET delivery from the nucleus to cell membrane. Second, *S. aureus* can also induce a slower, lethal form of NET release via the NADPH oxidase. The *S. aureus* Panton-Valentine Leukocidin (PVL) toxin initiates a third form of NET release in human neutrophils that is independent of the NADPH oxidase but depends on PAD4, MPO and histone citrullination, results in the death of the neutrophil and the release of NETs that have no antimicrobial potency. S. aureus protects itself against the attack of NETs by producing the Nuc1 nuclease that degrades the DNA backbone, by releasing the extracellular adherence protein (Eap) to inhibit NET-associated serine proteases and by generating fibronectin-binding protein B (FnBPB) to neutralize the bactericidal activity of histones. Extruded NETs have been proposed to promote *S. aureus* biofilm formation. CF neutrophils demonstrate delayed apoptosis and enhanced NET formation *in vitro*. *P. aeruginosa* has been described to interfere with the proliferation of *S. aureus* in mixed biofilms. Calprotectin, released by neutrophils, was reported to protect *S. aureus* from such actions executed by *P. aeruginosa*.

*S. aureus* also uses biofilms to evade the host immune system. One mechanism by which *S. aureus* evades the immune system is through the production of leukocidins, specifically Panton-Valentine leukocidin (PVL) and γ-Hemolysin AB (HlgAB), which induce formation of NETs in neutrophils that represents a mechanism ineffective at clearing bacteria within the biofilm (Bhattacharya et al., 2018). This was found to be true in a chronic wound infection model of *S. aureus*, suggesting that it could also apply to the chronic lung infection in CF (Bhattacharya et al., 2018). One non-CF study of *S. aureus* biofilm formation on catheters showed that biofilm formation inhibited the function of Toll-like receptor 2 (TLR2) and TLR9, reduced pro-inflammatory responses, and prevented phagocytosis by macrophages (Thurlow et al., 2011). Interestingly, the story may be different in CF biofilms. One report showed that the biofilms of two CF clinical isolates of *S. aureus* were better able to stimulate the production of the macrophage chemokines MIP-1a and MCP-1, the pro-inflammatory cytokines TNF-α, IL-6, and the anti-inflammatory cytokine IL-10, compared to the non-CF strains tested (Sadowska et al., 2013). This was corroborated by a second observation which reported that biofilm components from two CF clinical isolates were also strong inducers of proinflammatory cytokine production in macrophages and neutrophils (Ciszek-Lenda et al., 2023). Despite this inflammatory effect, however, both *S. aureus* strains were less inflammatory than their *P. aeruginosa* counterparts isolated from the same patient (Ciszek-Lenda et al., 2023). These studies give support to the idea that the CF lung exists in an inflammatory state, that is fueled in part by *S. aureus* biofilms. This inflammatory state is likely beneficial to the survival of *S. aureus* in the CF lungs, as calprotectin, a metal-chelating protein produced by neutrophils, has been demonstrated to promote the growth of *S. aureus* in co-species biofilms with *P. aeruginosa* (**Figure 2**) (Wakeman et al., 2016). Calprotectin performs this by attenuating quorum sensing and reducing the generation of anti-staphylococcal molecules of *P. aeruginosa* (Wakeman et al., 2016; Lee et al., 2025a). Whether metal sequestration is required for this mechanism of calprotectin, remains debated (Wakeman et al., 2016; Lee et al., 2025b). In a CF lung explant model, a *S. aureus* and *P. aeruginosa* biofilm was identified in an area of active inflammation and high calprotectin expression (Wakeman et al., 2016). Co-culture with *P. aeruginosa* may also assist *S. aureus* in avoiding phagocytosis as one study found that a co-species biofilm protected *S. aureus* from being phagocytosed by the amoeba *Dictyostelium discoideum* (Yang et al., 2011). It remains, however, unclear whether this applies to human phagocytes, as well. While significant literature exists regarding the role of *S. aureus* biofilms in antibacterial resistance and immune evasion, more future research is needed to study the role of *S. aureus* biofilms in the specific context of CF lung disease.

### 3.4 Virulence factors

Many of the virulence factors, especially exotoxins, carried by *S. aureus* are under the control of the accessory gene regulator (Agr) system (Jenul and Horswill, 2019). The *agr* locus contains two transcripts, RNAII which encodes the quorum sensing functionality of Agr, and RNAIII which regulates many target virulence genes (Jenul and Horswill, 2019). Among the virulence factors up-regulated by RNAIII expression are serine proteases SplA-F, alpha toxin, gamma hemolysin, and lipase (Dunman et al., 2001; Keitsch et al., 2018a; Jenul and Horswill, 2019). Agr expression also leads to the down-regulation of various surface proteins such as staphylococcal protein A (SpA), cell wall secretory protein, and surface receptors (MnhA, MnhF, and MnhG) (Dunman et al., 2001; Jenul and Horswill, 2019). The use of Agr in the pathogenesis of *S. aureus* seems to be a mechanism to shift from colonization to infection. Once a quorum is established and sensed, surface proteins are down-regulated while virulence factors are up-regulated. Agr quorum sensing is mediated by an autoinducing peptide (AIP), of which there are four main types that have been linked to different diseases (Novick, 2003). However, in CF the expression of all four AIP types is possible and none of them dominate (Kahl et al., 2003). Interestingly, in CF SCVs, the Agr system is down-regulated while SigB, and genes under the control of SigB, are up-regulated (Moisan et al., 2006). Another study examined pairs of *S. aureus* clones isolated from lung secretions of pediatric PwCF at early and later timer points, and found that in 6 of the 14 clonal pairs studied, the later isolates were less cytotoxic than the early isolates (Ramond et al., 2022). Additionally, less RNAIII expression was reported in all 6 of these later isolates, suggesting that Agr is down-regulated as *S. aureus* adapts to the CF lung environment (Ramond et al., 2022). However, the RNAIII expression of the remaining 8 clonal pairs were not investigated where the later isolates displayed the same or increased cytotoxicity as the early isolates (Ramond et al., 2022). Another work also determined low levels of RNAIII expression in the sputum of CF patients infected with *S. aureus* (Goerke et al., 2000). Co-infection with *P. aeruginosa* can also lead to decreased Agr expression as one report determined that *N*-acylhomoserine lactones (AHLs) produced by *P. aeruginosa* lead to decreased alpha toxin production, increased SpA production, and decreased RNAIII expression (Qazi et al., 2006). The down-regulation of Agr leads to an increase in SCV and biofilm formation, allowing the bacteria to persist in the CF lung (Goerke and Wolz, 2010). Overall, these data suggest that Agr is down-regulated as *S. aureus* adapts to the CF airway environment, which represents a mechanism to enhance persistence and avoid immune clearance by reducing expression of proinflammatory toxins (**Figure 1**).

Panton-Valentine leukocidin (PVL) is another virulence factor of *S. aureus* that plays an interesting role in disease progression. PVL is a two-component cytotoxin that targets neutrophils for lysis (Lo and Wang, 2011). *S. aureus* secrets the two components of PVL, LukS-PV and LukF-PV, into the environment where LukS-PV binds to the complement C5a receptor (C5aR) on the cell membrane of neutrophils (Nastaly et al., 2010; Spaan et al., 2013). LukS-PV then dimerizes with LukF-PV, eventually forming a heptamer which lyses the neutrophil (Nastaly et al., 2010). Additionally, PVL can induce apoptosis at low concentrations as opposed to triggering lysis at higher doses (Nastaly et al., 2010). PVL-positive *S. aureus* strains are still relatively rare in CF. Out of 88 clinical isolates of *S. aureus* from various diseases investigated, only two produced PVL and only one of those was a CF isolate (Cakır Aktas et al., 2013). Only 8.6% of CF clinical isolates were identified as PVL-positive in another study (Rodrigues et al., 2020). An analysis from a children’s hospital in Italy revealed that PVL expression was less common among *S. aureus* isolates from the CF unit than among those originating from other units as only two CF isolates were found to express PVL (Manara et al., 2018). Even if it is rare, PVL-positive *S. aureus* infection leads to severe disease. A case report from the United Kingdom described a male CF patient in his 20’s who first reported with dermatological infection on his face and was given antibiotics only to return two weeks later with a severe pulmonary exacerbation (Barry et al., 2014). This was characterized by a 5% decrease in weight, a 20% decrease in spirometry measures, leukocytosis, hemoptysis, and tachycardia, among other symptoms (Barry et al., 2014). The analysis of both skin swabs from the initial infection and sputum from the exacerbation revealed a PVL-positive MSSA strain (Barry et al., 2014). However, another publication across 7 different CF clinics across the United States could not identify a difference in lung function or nutrition levels based on PVL status of *S. aureus* isolates (Heltshe et al., 2015). PVL-positive *S. aureus* may also be passed from one CF patient to another. In support of this, one hospital in the United States reported two separate incidents in which pediatric CF individuals reported to the hospital with acute respiratory failure requiring intubation due to PVL-positive MSSA infections acquired from their sibling who also had CF (Elizur et al., 2007). In both cases, the older sibling tested positive for PVL-positive MSSA before passing it to their younger, more susceptible sibling (Elizur et al., 2007). Therefore, it remains unclear exactly what role PVL plays in *S. aureus* infections of CF airways.

A common problem of *S. aureus* infection both in PwCF and in non-CF individuals is the reoccurrence of the infection, indicating that previous infection does not induce complete immunological protection. This is in part due to the function of a protein expressed on the surface of *S. aureus* known as staphylococcal protein A (SpA). One known mechanism by which SpA aids *S. aureus* in avoiding the immune system is its ability to bind the Fc region of IgG antibodies due to the presence of 4 or 5 Ig binding domains (Kim et al., 2012). This allows *S. aureus* to inhibit opsonophagocytosis by neutrophils as the antibodies are not recognized by neutrophilic Fc receptors. SpA can also bind to certain Fab regions of B cell receptors, particularly those of the VH3 class, eventually leading to a depletion of the B cell repertoire (Goodyear and Silverman, 2004). In CF, SpA expression may be up-regulated as Agr is down-regulated (**Figure 1**). A report comparing early and late *S. aureus* isolates in pediatric CF patients found that SpA expression was up-regulated in late isolates and associated with down-regulation of Agr expression (Ramond et al., 2022). In addition to being found on the cell surface, SpA can also be released into the environment. Supporting this, the laboratory strain USA300 was reported to release more SpA into the extracellular matrix than two CF isolates of *S. aureus* (Ludwig et al., 2023). This may be a mechanism of immune evasion as they also found that infection of neutrophils by the two CF isolates resulted in less reactive oxygen species (ROS) production, compared to neutrophils infected with USA300 (Ludwig et al., 2023). SpA also likely gives *S. aureus* an advantage in the CF lung during co-infections with *P. aeruginosa*, as SpA reduces the ability of CF isolates of *P. aeruginosa* to produce biofilms (**Figure 1**) (Armbruster et al., 2016). This was speculated to be due to the ability of SpA to bind to the *P. aeruginosa* polysaccharide PsL as well as PilA, a component the type IV pili (Armbruster et al., 2016). Interestingly though, SpA could also be beneficial to *P. aeruginosa* in co-infection as it was found to protect *P. aeruginosa* from being phagocytosed by neutrophils by binding to the Fc region of IgG, the same mechanism by which it protects *S. aureus* from phagocytosis (Armbruster et al., 2016). *S. aureus* isolates can be characterized by their SpA type which is determined by the number of short sequence repeats (SSR) found in the Xr region of the gene (Shopsin et al., 1999). *S. aureus* isolates with a fewer number of SSRs result in less IFN-β production and less neutrophil recruitment *in vivo* (Garofalo et al., 2012). Further, *S. aureus* isolates from chronic infections, including CF, tended to have fewer SSRs, while those from acute infections had more, providing more evidence to the immune evasion role of SpA in CF (Garofalo et al., 2012). The roles of SpA in *S. aureus* immune evasion are complex, but it appears that SpA plays an important part in infection in CF.

## 4 Neutrophil response to *S. aureus* in CF

Neutrophils are the primary cellular component of the innate immune system, comprising 40-60% of all white blood cells in the blood. As a primary phagocyte, their role is to defend the host against bacterial infections. Neutrophils are required for the elimination of bacterial infections, including *S. aureus*. This is evidenced by the fact that neutrophil deficiencies often result in increased occurrence or severity of *S. aureus* infection (Liu et al., 2018; Kurz et al., 2024). One study reported that 99% of infections in patients with congenital neutropenia, a disease characterized by reduced neutrophil numbers in the blood, were due to bacterial origins (Rotulo et al., 2021). Further, of those bacterial infections, *S. aureus* was the most common, accounting for 38% of all cases (Rotulo et al., 2021). Mice with a mutation in the granulocyte colony stimulating factor receptor (G-CSFR) gene (*Csf3*), encoding a key protein in the development of neutrophils, demonstrated increased mortality following *S. aureus* infection, compared to wild-type control mice (Mitsui et al., 2003). Additionally, individuals with mutations in TLR genes, many of which are found on neutrophils, suffered from increased susceptibility to skin infections, primarily *S. aureus* (Stappers et al., 2014). Mice without functional TLR2 as well as deficient in other cytokines and chemokines like TNF-α presented with increased bacterial load and decreased neutrophilic inflammation in response to *S. aureus* corneal infections (Callegan et al., 2025). This indicates a critical role of neutrophils in response to staphylococcal keratitis. Neutrophils are also important to control the spread of *S. aureus* skin infections, as mice without functional neutrophil elastase (NE) suffered more severe skin infections compared to control mice (Kanoza et al., 2025). However, the neutrophilic response to *S. aureus* infection needs to be kept in balance as an increase in the neutrophil to lymphocyte ratio in the blood is an indicator of increased risk of death during *S. aureus* bacteremia (Greenberg et al., 2018; Özdemir et al., 2025). Overall, these data indicate that neutrophils are required for a proper immune response to *S. aureus*, no matter where the infection occurs (lungs, skin, cornea, or blood) or in humans or mice. The following sections will briefly discuss how neutrophils kill *S. aureus* in people without CF and will go more in depth on neutrophil/*S. aureus* interactions in PwCF. For a more detailed, general summary of *S. aureus* killing by neutrophils or proposed differences of CF neutrophils and healthy neutrophils, we refer the reader to other, excellent sources (McGuinness et al., 2016; Buvelot et al., 2017; Kobayashi et al., 2018; Rungelrath and DeLeo, 2021; Patel and Nugent, 2023).

### 4.1 Neutrophil killing of *S. aureus* in people without CF

In principle, neutrophils can kill bacteria by two main mechanisms: 1) intracellular, phagosomal killing or 2) extracellular killing mediated by neutrophil extracellular traps (Brinkmann et al., 2004). When microbes are killed intracellularly, neutrophil receptors recognizing microbial molecules (pathogen-associated molecular patterns) or host molecules deposited on the microorganism (opsonins) will bind to their ligand and initiate bacterial uptake. After phagocytosis, granules of the neutrophils fuse with the phagosome, forming the phagolysosome, and exposing the bacterium to an arsenal of antimicrobial molecules leading to its destruction (Rada et al., 2008). Microbes can also be killed extracellularly when neutrophils expel NETs in response to the pathogen challenge (Brinkmann et al., 2004). NETs entrap the pathogens, and histones and granule enzymes associated with NETs result in entrapment and killing of the bacterium. Antimicrobial activities of neutrophils can also be categorized into oxidative and non-oxidative mechanisms, based on the requirement of oxygen to generate reactive oxygen species (McGuinness et al., 2016).

The main mechanism by which neutrophils kill *S. aureus* is phagocytosis and related oxidative, intracellular killing. This is mainly evidenced by the frequent *S. aureus* infections observed in people with chronic granulomatous disease (CGD), a condition characterized by a defective NADPH oxidase (Buvelot et al., 2017; Justiz-Vaillant et al., 2023). *S. aureus* can cause infections practically in any human host, but in individuals with a functional NOX2-based NADPH oxidase, *S. aureus* infections are infrequent and primarily resort to the skin (Buvelot et al., 2017). In contrast, in CGD patients, *S. aureus* readily survives and often causes clinical disease (Buvelot et al., 2017). *S. aureus* is the most common organism isolated from CGD patients (Marciano et al., 2015; Buvelot et al., 2017). In a mouse model of CGD, neutrophils deficient in the NADPH oxidase subunit p40^phox^ displayed decreased ability to kill *S. aureus* both *in vitro* and *in vivo* (Ellson et al., 2006).

Oxygen-dependent bacterial killing primarily relies on the function of the NADPH oxidase, which is assembled and activated upon phagocytosis of a bacterium (Nunes et al., 2013; Winterbourn and Kettle, 2013) (**Figure 2**). *In vitro S. aureus* killing by human neutrophils is entirely inhibited by an irreversible inhibitor of the NADPH oxidase, and other flavoenzymes, diphenyleneiodonium chloride (DPI), while *E. coli* killing remains largely unaffected (Rada et al., 2004). The NADPH oxidase produces superoxide anions (O_2_^-^) by transporting electrons from the cytoplasm into the phagosome and donating them to molecular oxygen (Winterbourn and Kettle, 2013; Nauseef, 2014a). O_2_^-^ is then converted to hydrogen peroxide (H_2_O_2_) spontaneously or with the help of superoxide dismutase (McGuinness et al., 2016). The conversion of O_2_^-^ to H_2_O_2_ requires the incorporation of protons (Kettle et al., 2023). The hydrogen ion is provided by a voltage-gated proton channel (Hv1) that extrudes protons derived from the NOX2-dependent consumption of NADPH in the cytosol into the phagolysosomal lumen (El Chemaly and Demaurex, 2012) (**Figure 2**). The Hv1 proton channel keeps the pH of the neutrophil phagosome initially at close-to-neutral values required for optimal microbial killing and also maintains a physiological phagosomal membrane potential essential for continued NADPH oxidase activity (**Figure 2**) (Henderson et al., 1988; DeCoursey et al., 2003; El Chemaly and Demaurex, 2012). H_2_O_2_ is next converted to hypochlorous acid (HOCl) through the action of myeloperoxidase (MPO) that uses Cl^-^ as a substrate (Winterbourn and Kettle, 2013). While some phagosomal Cl^-^ could be captured from the extracellular environment during phagocytosis (depending on the tightness of the forming phagosomal membrane around the microbe), Cl^-^ is mainly thought to be transported into the phagosome from the cytosol via transporters. Several transporters have been proposed to enrich the phagosome with Cl^-^, including ClC3, KCC3, and CFTR (**Figure 2**) (Moreland et al., 2006; Painter et al., 2006; Sun et al., 2012; Foote et al., 2017). Most likely, each of them contributes to the overall intraphagosomal Cl^-^ transport required for optimal phagosomal bacterial killing. Interestingly, MPO-deficient humans are largely asymptomatic and only have higher incidence rates of certain fungal infections (Nauseef, 2014b). Since a large body of data suggests that HOCl is microbicidal against most microbes *in vitro*, HOCl generation takes place in the neutrophil phagosome and both, microbial and host proteins are chlorinated by phagosomal HOCl, *in vivo* MPO likely represents a first line of defense against a wide variety of microbial intruders but redundant, secondary mechanisms are in place, in case MPO fails or is insufficient (Boecker et al., 2023).

As part of the antioxidant shield of *S. aureus*, the bacterium uses the *katA* catalase to degrade neutrophil-generated H_2_O_2_. KatA reduces oxidative stress and subsequent DNA damage in *S. aureus in vitro* (Cosgrove et al., 2007; Linzner et al., 2022).This catalase is required for enhanced survival in human neutrophils *in vitro* (**Figure 2**), and *in vivo* survival and persistence of *S. aureus* in a nasal colonization cotton rat model (Cosgrove et al., 2007).

H_2_O_2_, HOCl and other reactive oxidants are major contributors to the bactericidal activity of neutrophils. While oxidant-dependent mechanisms are crucial for neutrophils to fight *S. aureus*, neutrophils also utilize a variety of oxygen-independent methods of bacterial killing, including serine proteases such as neutrophil elastase (NE), cathepsin G (CatG), and proteinase 3 (PR3) (McGuinness et al., 2016; Mall et al., 2024). Overall, *S. aureus* infections are characteristic in CGD patients but not in patient with MPO deficiency, emphasizing the essential nature of the NADPH oxidase and ROS, but not necessarily HOCl, in *S. aureus* elimination by neutrophils in humans.

In addition to phagocytosis, neutrophils can also kill invading bacteria through the production of NETs (Brinkmann et al., 2004). NETs are comprised of a DNA scaffold that is studded with histones and granule proteins such as NE, CatG, PR3, and MPO (Brinkmann et al., 2004). NET production occurs by two major mechanisms, lytic and non-lytic processes (Delgado-Rizo et al., 2017; Wang et al., 2024). Lytic or suicidal NETosis can be induced by the binding of bacteria or antibodies to neutrophil receptors resulting in the activation of the NADPH oxidase (Delgado-Rizo et al., 2017; Wang et al., 2024). This leads to the activation of NE which results in the degradation of actin, citrullination of histones by protein arginine deiminase 4 (PAD4), the breakdown of the nuclear membrane, and the eventual release of chromatin by cell lysis (Delgado-Rizo et al., 2017; Wang et al., 2024). Non-lytic NET generation is induced by the binding of TLRs or complement C3 receptor and is NADPH oxidase-independent (Delgado-Rizo et al., 2017; Wang et al., 2024). Instead of being released by cell lysis, chromatin is released through vesicular transport, allowing the denucleated neutrophil to continue to perform chemotaxis and phagocytosis (Delgado-Rizo et al., 2017; Wang et al., 2024). Assays to detect or quantify NET production typically involve staining extracellular DNA, citrullinated histone H3, or other proteins often associated with NETs such as NE or MPO and detection with ELISA, immunofluorescent microscopy, or Western blot (Stoimenou et al., 2022). NETs are capable of trapping both Gram-positive and Gram-negative bacteria, making them an important member of the neutrophil antibacterial arsenal (Brinkmann et al., 2004).

When exposed to human serum, *S. aureus* creates a thicker cell wall, leading to impaired opsonization and decreased killing by neutrophils, providing additional evidence to the importance of intracellular *S. aureus* killing (Ledger and Edwards, 2024). Work from our team has also shown that cytochalasin-B, an inhibitor of the cytoskeleton and phagocytosis, inhibited killing of MRSA by human neutrophils (Fantone et al., 2023). However, when neutrophils were treated with extracellular DNase to degrade NETs, there was no impairment in *in vitro* MRSA killing (Fantone et al., 2023). NETs seem to be less effective at killing *S. aureus* as the bacterium possesses several mechanisms to neutralize their effectiveness (Speziale and Pietrocola, 2021). These factors include nucleases which degrade the DNA backbone, the extracellular adherence protein Eap which inhibits neutrophil serine proteases, and fibronectin-binding protein B (FnBPB) which blocks the bactericidal activity of histone H3 (Speziale and Pietrocola, 2021). Nuclease-dependent degradation of NETs may play an important role in *S. aureus* lung infections as mutant strains unable to produce the nuclease demonstrated decreased bacterial load and subsequent increased survival of intranasally infected mice, compared to wild-type, nuclease producing bacteria (Berends et al., 2010). PVL, and other *S. aureus*-derived leukocidins, induce NET formation resulting in decreased bacterial killing (**Figure 2**) (Bhattacharya et al., 2018). Interestingly, PVL-induced NETs have a different composition compared to NETs induced by other stimuli, including a lower concentration of NE and PR3, which may explain their decreased ability to kill *S. aureus* (Jhelum et al., 2023). PVL-induced NETs are decorated with NE and citrullinated histones and their formation depends on mitochondria and MPO but not the NADPH oxidase (**Figure 2**) (Mazzoleni et al., 2021). Furthermore, in a mouse model of infective endocarditis, it was found that neutrophils were crucial for controlling *S. aureus* burden, however mice impaired in PAD4-mediated NET formation, did not present increased bacterial burden compared to control mice (Meyers et al., 2023). Overall, neutrophils primarily kill *S. aureus* through intracellular, oxidative killing mechanisms and extracellular *S. aureus* killing by NETs is less effective.

### 4.2 Neutrophil killing of *S. aureus* in PwCF

The frequency of certain bacterial infections despite robust neutrophilic inflammation in the lungs of PwCF suggests a defect in the bactericidal ability of neutrophils in CF. Some studies propose an endogenous, intrinsic role of CFTR in neutrophils to kill bacteria including *P. aeruginosa* (Painter et al., 2008; Ng et al., 2014; Dickerhof et al., 2019; Patel and Nugent, 2023; Da Silva Cunha et al., 2024). CFTR has been proposed to deliver Cl^-^ into the phagosome to fuel microbicidal HOCl production (**Figure 2**) (Painter et al., 2006; Aiken et al., 2012; Ng et al., 2014). This is based on results of experiments performed using human or murine primary neutrophils that were deficient in *CFTR/Cftr* or were treated with CFTR inhibitors (Hayes et al., 2020; Da Silva Cunha et al., 2024). Prolonged phagosomal acidification and impaired bacterial killing were observed in human CF neutrophils or control neutrophils treated with the CFTR inhibitor, CFTRinh-172 (Hayes et al., 2020; Da Silva Cunha et al., 2024). One study found that the anti-staphylococcal defect of CF neutrophils was not due to an inability to phagocytose, as both healthy and CF neutrophils phagocytosed *S. aureus* to the same degree, but rather to altered degranulation and pH of the phagosome, which was measured by staining the bacteria with a pH sensitive dye (Hayes et al., 2020). CF phagosomes displayed an increased pH of 8 at 8 minutes post-phagocytosis compared to a pH of 7.3 in healthy phagosomes (Hayes et al., 2020). This increase in phagosomal pH was lost when the patient was on ivacaftor, a CFTR modulator therapy, suggesting a role of the mutated CFTR gene in neutrophil dysfunction in CF (Hayes et al., 2020).

Other members of the lung microbiome may also affect the ability of neutrophils to kill *S. aureus*. Previous exposure of mice to *Prevotella melaninogenica*, a Gram-negative anaerobe, resulted in increased neutrophilic killing of *S. aureus* (Goryachok et al., 2025). Interestingly, this may also be CFTR-dependent as CFTR-deficient bronchial epithelial cells demonstrated less *S. aureus* attachment, resulting in increased bacterial burden, regardless of previous exposure to *P. melaninogenica* (Goryachok et al., 2025). However, the protective nature of *P. melaninogenica* was restored following treatment with the CFTR modulators elexacaftor and tezacaftor (Goryachok et al., 2025). The finding that *P. melaninogenica* exposure may increase the ability of neutrophils to kill *S. aureus* suggests a role for trained immunity in CF, however this requires further investigation.

Very few reports have investigated *S. aureus* killing by neutrophils in the context of CF. Our studies reported no impairment in the ability of human CF neutrophils to kill methicillin-sensitive or -resistant *S. aureus* CF isolates *in vitro* (Fantone et al., 2021; Fantone et al., 2023). CF neutrophils phagocytosed MRSA with the same efficacy as control subjects’ neutrophils (Fantone et al., 2023). Interestingly, neutrophil-mediated killing of only one reference bacterial strain, USA300, was affected by CFTR deficiency of the neutrophils (Fantone et al., 2021; Fantone et al., 2023). These data emphasize the importance of including clinically more relevant CF isolates and the need for testing a large number of clinical isolates. NETs have been documented in CF airways and NET formation has been proposed to contribute to CF lung disease (Gray et al., 2015; Fantone et al., 2025). NET formation has been shown to be affected by CFTR as neutrophils from CFTR-deficient piglets and PwCF showed a pro-survival phenotype and a delay in apoptosis which led to increased NET release (**Figure 2**) (Gray et al., 2018). This pro-survival phenotype was reversed when the neutrophil donor was taking ivacaftor (Gray et al., 2018). As in people without CF, NETs also seem less effective at killing *S. aureus*, including CF clinical isolates (Fantone et al., 2023). This may be due to increased nuclease activity of the bacterium, as CF clinical isolates of *S. aureus* with high nuclease activity demonstrated increased survival when exposed to NETs (Herzog et al., 2019). Research from our own laboratory has also shown nuclease activity in CF isolates of both MSSA and MRSA, however there was no correlation between NET release induced by the *S. aureus* isolates and their nuclease activity (Fantone et al., 2021). Therefore, CFTR deficiency leads to increased NET formation which could result in increased survival of *S. aureus* isolates that have increased nuclease activity.

Additionally, it has been proposed that CFTR-deficient neutrophils are less able to transport chloride into the phagolysosome, affecting the ability of the neutrophil to chlorinate bacteria with HOCl (Painter et al., 2006; Painter et al., 2010). This may lead to the selection of *S. aureus* and *P. aeruginosa* in CF as these bacteria require higher concentrations of HOCl to be killed than non-CF pathogens such as *Escherichia coli* and *Klebsiella pneumoniae* (Jennings et al., 2023). Additionally, mice with both global and myeloid-lineage specific CFTR deficiency had increased *S. aureus* burden in their BALF compared to control mice (Jennings et al., 2023). Interestingly, both *P. aeruginosa* and *S. aureus* outcompeted the non-CF pathogens in both CF mouse models despite an initial lower dose at inoculation, which the authors contributed to their higher resistance to HOCl-mediated killing (Jennings et al., 2023). CFTR does not seem to affect the speed of neutrophil recruitment as CF neutrophils were recruited to *S. aureus* particles quicker than non-CF neutrophils (Yonker et al., 2021). CF neutrophils also formed larger phagocytosis aggregates than non-CF neutrophils (Yonker et al., 2021). This was dependent on the disease status of the donor as neutrophils from hospitalized PwCF experiencing an exacerbation formed larger aggregates than neutrophils from outpatient PwCF (Yonker et al., 2021). The concentration of NaCl of the airway surface liquid does not affect the ability of neutrophils to kill *S. aureus* (Verghese and Boucher, 1998). Additionally, the high oxidative stress environment of CF sputum causes an increase in the expression of superoxide dismutases A and M (SodA/M) in *S. aureus* (Treffon et al., 2020). SodA and SodM scavenge superoxide anions formed by the NADPH oxidase in the phagosome and protect bacteria from neutrophilic oxidative killing (Treffon et al., 2020). Interestingly, it seems as though SodA and SodM are complimentary as only bacteria deficient in both of them demonstrated a significant survival disadvantage when exposed to neutrophils, compared to the control and single knockout (Treffon et al., 2020). Overall, these data indicate a role for CFTR deficiency in neutrophil dysfunction and *S. aureus* persistence in CF lung disease.

Although mutations in CFTR have been shown to affect neutrophil function, lung infections in PwCF rarely spread to other sites and PwCF are not at increased risk of systemic infections, suggesting that endogenous CFTR deficiency isn’t the sole cause for neutrophil dysfunction in CF. Our research has shown that peripheral blood neutrophils from PwCF are able to kill both MSSA and MRSA clinical isolates *in vitro* to the same degree as peripheral blood neutrophils from age- and sex-matched, healthy individuals (Fantone et al., 2021). Interestingly, CF neutrophils were only deficient at killing the laboratory strain USA300 (Fantone et al., 2021). Therefore, it is likely that unknown components present in the CF lung microenvironment, rather than the intrinsic CFTR mutation, are affecting the ability of CF neutrophils to kill *S. aureus*. Our work showed that when non-CF human neutrophils were exposed to the supernatant of sputum from PwCF, as a model for the CF lung microenvironment, their ability to kill *S. aureus* was significantly reduced (Fantone et al., 2021). However, the ability of neutrophils to attach to and phagocytose *S. aureus* was not affected (Fantone et al., 2021). Further investigation showed that exposure of healthy neutrophils to CF sputum reduced phagosomal ROS levels following phagocytosis of MRSA, while total ROS production was not affected (Fantone et al., 2023). Total ROS production was measured using a Diogenes-based chemiluminescence kit while phagosomal ROS was measured using imaging flow cytometry (Fantone et al., 2021; Fantone et al., 2023). Assessing phagosomal, but not cytosolic or extracellular, ROS production is very important as *S. aureus* is killed by ROS produced in the well-defined niche of the phagosome, not in other compartments. Interestingly, localization of MPO and CatD, but not NE, to the phagolysosome was also reduced upon exposure of neutrophils to CF sputum (Fantone et al., 2023). These results indicate that the CF airway environment limits the fusion of phagosome with primary granules that could affect the bacterial killing process in neutrophils (**Figure 2**). It remains a largely open question whether the CF lumen also has similar effects on other granule subsets of neutrophils such as secondary and tertiary granules, not only primary granules (**Figure 2**).

In order to identify what component of the CF sputum causes reduced neutrophilic killing of *S. aureus*, we exposed healthy neutrophils to short chain fatty acids (SCFAs) which are found in millimolar concentrations in the CF airways as a metabolic byproduct of anaerobic bacteria (Miller et al., 2023). SCFA exposure did not affect NET release but did reduce ROS production upon stimulation with MRSA (Miller et al., 2023). However, treatment of neutrophils with SCFAs did not significantly reduce their ability to kill MRSA (Miller et al., 2023). Since neutrophils pretreated with healthy sputum did not display any significant reduction in *S. aureus* killing, this suggests a scenario in which the neutrophil’s ability to phagocytose and kill *S. aureus* is dependent on the presence of CF sputum. The makeup and the contents of the sputum could give reason as to why there is reduced clearance of *S. aureus* by neutrophils when introduced to the lung environment.

Additional effects of the CF airway environment on the function of neutrophils have also been described. While these studies did not typically address the ability of neutrophils to kill *S. aureus*, it is important to speculate the consequences of their findings for *S. aureus* killing in CF. Viable but dysfunctional neutrophils were detected in CF airways that show increased expression of primary granule marker CD63 and decreased expression of phagocytic receptors such as CD16 and CD14 (Tirouvanziam et al., 2008). One component of the CF lung microenvironment that may play a role in neutrophil dysfunction are extracellular vesicles (EVs), including exosomes, which are produced in part by neutrophils (Genschmer et al., 2019). Exosomes are small vesicles released by neutrophils, as well as many other cell types, that tend to take on the characteristics of the cell in which they were derived from by containing intracellular content pertaining to the origin cell type (Kawabata et al., 2002; Kalluri and LeBleu, 2020). This can include a wide variety of cargo such as nucleic acids, proteins, lipids, proteases, metabolites, etc (Kalluri and LeBleu, 2020; Kumari et al., 2024). This allows exosomes to play a role in further eliciting the immune response through critical cell-to-cell communication (Di Gioia et al., 2022; Lee et al., 2024). Exosomes also have the ability to carry NE, which can bind to and degrade the extracellular matrix as well as MPO which produces strong antimicrobial oxidants such as HOCl that can also destroy host tissues (Genschmer et al., 2019; Xie et al., 2022; Ben-Meir et al., 2024). Because CF is characterized by excessive dehydrated mucus clogging the airways, exosomes are often found in high abundance in the airways of PwCF which can lead to elevated concentrations of NE and MPO present in sputum (Fantone et al., 2021).

Another factor to consider for the lack of *S. aureus* clearance by neutrophils in the CF lung environment is the potential for the increase in production of NETs and mitochondrial exchange by neutrophils due to the release of EVs. The activation of platelets can cause the release of EVs that contain respiration-competent mitochondria within them (Allan et al., 2025). When neutrophils engulf these EVs, it can induce an activated state, and the mitochondria can cause neutrophil function to change (Allan et al., 2025). Activation of the neutrophil by mitochondrial uptake caused an increase in NET production, with or without external stimuli applied (Allan et al., 2025). The increase in NET formation led to a reduction in the ability of neutrophils to phagocytose *E. coli*, showing a decrease in the phagocytic ability of the neutrophil (Allan et al., 2025).

Although only correlative results from the blood, our data indicate a significant and strong association between the high systemic IgM autoantibody profile and the absence of *S. aureus* lung infection in adult PwCF (Yadav et al., 2023). No such results were seen with IgA, IgG or IgE autoantibodies (Yadav et al., 2023). These data propose the interesting hypothesis that IgM autoantibodies developed against host molecules could somehow help *S. aureus* clearance in CF. While several potential mechanisms could be proposed, many including neutrophils, they remain speculative at this point and additional studies using more CF cohorts are required to confirm this observation and to investigate its mechanism (Yadav et al., 2023).

Overall, it is most likely that a combination of both intrinsic CFTR deficiency in neutrophils and several environmental factors of the CF lung (EVs, NETs, antibodies, SCFAs, cytokines, microbial molecules … etc.) contribute to the reduced ability of CF neutrophils to kill *S. aureus*.

## 5 *In vivo* models of *S. aureus* lung infection in CF

*In vivo* models are especially important to understand the mechanisms of neutrophil response to *S. aureus* infection in the CF lung, as many cell types in the lung rely on appropriate CFTR function. To date, most *in vivo* infections of *S. aureus* have employed rodent (mice or rat) models, which will be discussed in detail.

### 5.1 Mice

Mouse models to understand pulmonary infections with *S. aureus* in CF have employed a number of mouse genotypes and *S. aureus* strains. Seminal work with the original CF mouse created, the CFTR^tm1UNC^ line, found that the development of pathology in response to *S. aureus* was heterogeneous (Snouwaert et al., 1995). In contrast, *Cftr^-/-^* mice were found more susceptible to clinical isolates of *S. aureus*, resulting in a larger lung burden acutely, but were unable to assay chronic infection (Cressman et al., 1998). Similar to the original infection described, the authors did not evaluate specific aspects of the immune response.

More recently, the field has turned to identification of the innate immune components that are active following *S. aureus* infection. The cytokine response to *S. aureus* in the *Cftr^-/-^* and WT mice has been carefully delineated, with and without gentamicin (Li et al., 2017). This work found that CF mice had decreased survival after *S. aureus* infection, until 5 days after exposure to a non-CF sepsis clinical isolate, and to the lab reference Newman strain. Although this group focused on macrophage phagocytosis of the *S. aureus* strains, they also noted that neutrophilia is a typical CF response to *S. aureus* infection in the lung. The innate immune response has been further characterized by the use of a *Mek2^-/-^* mouse, which found that 24 hours after infection with USA300 the response to *S. aureus* infection is primarily neutrophilic (Zuiker et al., 2025). In addition, the CF mouse model has been used by investigators to understand the pathology of the bacteria directly. For instance, α-toxin was found to be required for lung infection of *S. aureus* (Keitsch et al., 2018b).

These studies demonstrate that the mouse models are useful, but have some important limitations, namely, the ability to understand the innate/host response and the bacterial pathology during chronic infections, which are more common to people with CF. Additionally, although the CF lung is characterized by severe and prolonged neutrophilia, prior to and in response to *S. aureus* infection, much of the existing literature using the mouse models have focused on the response of macrophages, not neutrophils (Guilbault et al., 2007).

### 5.2 CF rats

The use of the CF rat models have attempted to fill this gap in difference between acute and chronic infection with *S. aureus*. Initial infections in the CFTR^-/-^ rat model have found that there are differences in *S. aureus* response between the CF and WT rats during acute timepoints, primarily by infected with a CF clinical isolate of *S. aureus*, SA0831 (Bollar et al., 2022). This manuscript also found important differences in the neutrophilic response to SCVs of this strain. Neutrophils were increased in the BALF of rats infected with SCVs, a phenotype of *S. aureus* that is also common in PwCF (Bollar et al., 2022). Follow-up studies found that exposure to the CFTR modulator ivacaftor resulted in spontaneous SCV generation in the CF rat lung, even during acute infection, raising the question of the SCV as an overlooked phenotype of importance in this population (Bollar et al., 2024). Addressing the limitation in the mouse models, the CF rat has been used to support chronic infections with *S. aureus*, using the SA0831 clinical isolate and laboratory reference strain JE2, alone in combination with *Pseudomonas aeruginosa* strains, again reinforcing the importance of the neutrophil response in the CF lung (Bollar et al., 2025).

## 6 Unanswered questions

Many strides have been made in the field of CF research which have resulted in the median predictable survival age of PwCF more than doubling from 30 years old in 1990 to 65 years old in 2024 (Cystic Fibrosis Foundation, 2025). However, there are still major gaps in the field, particularly when it comes to the interactions of *S. aureus* and neutrophils. First, the exact effect of *S. aureus* infection on CF lung disease, particularly in adults, still remains uncertain. While some studies found that *S. aureus* infection was associated with decreased lung function, including measures such as FVC and FEV_1%_, others found that *S. aureus* infection resulted in fewer exacerbations and that *P. aeruginosa* infection resulted in worse outcomes (Wolter et al., 2013; Ahlgren et al., 2015; Kussek et al., 2022; Sunman et al., 2022; Thornton et al., 2023). While *P. aeruginosa* is the more prominent bacterial pathogen in adult PwCF, it is still important to know the exact effects of *S. aureus* infection as it affects up to 30% of adult PwCF above the age of 30 (Cystic Fibrosis Foundation, 2025). Increased monitoring for infection and lung function among large cohorts of patients can help answer this question.

Additionally, as multiple bacterial pathogens are often found in the CF lung, it’s important to understand how *S. aureus* interacts with other bacteria in CF. As stated above, co-infection of *S. aureus* with *P. aeruginosa* may lead to enhanced survival of *S. aureus* due to the formation of SCVs, increased expression of SpA, decreased Agr expression, and increased antibiotic resistance in co-species biofilms (Hoffman et al., 2006; Qazi et al., 2006; Armbruster et al., 2016; Orazi and O’Toole, 2017). However, it is unclear how *P. aeruginosa* and *S. aureus* co-species biofilms affect phagocytosis by neutrophils and other human phagocytes as the only study on the matter used the amoeba *Dictyostelium discoideum* as a phagocyte (Yang et al., 2011).

Many of the studies investigating neutrophil dysfunction in CF are limited by only studying the effects on killing of *P. aeruginosa* and not *S. aureus*. As *P. aeruginosa* is a gram-negative bacterium and *S. aureus* is a gram-positive, it is illogical to assume that the same findings apply to both species. More studies are needed specifically investigating the effects of CFTR dysfunction on neutrophilic killing of *S. aureus.* Additionally, more investigation into the composition of CF sputum is needed to understand how the microenvironment of CF airways affects neutrophilic killing of *S. aureus* (Ben-Meir et al., 2024).

It is also unclear what is the exact role that PVL plays in *S. aureus* pathogenesis in CF. While still relatively rare in CF, one case study showed that PVL-positive *S. aureus* infection resulted in increased lung disease while another survey found no correlation between PVL-positive *S. aureus* infection and decreased lung function in CF (Barry et al., 2014; Heltshe et al., 2015; Rodrigues et al., 2020). As PVL can target and kill neutrophils, understanding its role in CF can provide a potential target for future therapies to improve clinical outcomes.

By addressing these remaining unanswered questions, we can better understand how *S. aureus* survives in CF, how neutrophils respond to *S. aureus* challenge, and how CFTR deficiency and the microenvironment interact to affect neutrophilic killing of *S. aureus.* This will aid in reducing the burden of *S. aureus* infection in PwCF and contribute to not just increased life expectancy, but improved quality of life, as well.

## 7 Concluding remarks

In summary, *S. aureus* remains a prevalent and resilient pathogen in CF, persisting despite intense immune responses from host neutrophils. Its survival in the CF lung is driven by a complex interplay of bacterial adaptations, such as biofilm formation, and defects of the host’s innate immune system, such as impaired CFTR function and ineffective NETs. Future research should utilize multi-omics analytical (proteomics, metabolomics, lipidomics) approaches to study airway biospecimen collected from PwCF for a better, more complex understanding of lung infections and inflammation. The development and validation of novel, *ex vivo* experimental models using human cells and clinical samples from PwCF are needed to better predict clinical outcomes of novel treatments and to personalize future therapies. Standardized genotyping frameworks are also required for bacterial transmission studies. Novel, targeted anti-biofilm therapeutic strategies are urgently needed to treat *S. aureus* biofilm infections in CF. Ultimately, bridging the gap in our understanding of *S. aureus* persistence and *S. aureus*-neutrophil interactions in the CF lung is essential for developing more effective treatments to target *S. aureus* in CF airways and to achieve improved clinical outcomes.
